# New clinical and molecular insights on Barth syndrome

**DOI:** 10.1186/1750-1172-8-27

**Published:** 2013-02-14

**Authors:** Lorenzo Ferri, Maria Alice Donati, Silvia Funghini, Sabrina Malvagia, Serena Catarzi, Licia Lugli, Luca Ragni, Enrico Bertini, Frédéréc M Vaz, David N Cooper, Renzo Guerrini, Amelia Morrone

**Affiliations:** 1 Department of Neurosciences, Psychology, Pharmacology and Child Health, University of Florence and Paediatric Neurology Unit and Laboratories, Meyer Children’s Hospital, Viale Pieraccini n. 24, 50139, Florence, Italy; 2 Metabolic and Muscular Unit, Neuroscience Department, Meyer Childrens’ Hospital, Florence, Italy; 3 Paediatric Neurology Unit and Laboratories, Neuroscience Department, Meyer Children’s Hospital, Florence, Italy; 4 Neonatology Unit, Department of Mother & Child, University of Modena, Modena, Italy; 5 University of Bologna and Azienda Ospedaliera S. Orsola Malpighi, Bologna, Italy; 6 Department of Neurosciences, Laboratory of Molecular Medicine, Bambino Gesu’ Children’s Research Hospital, Rome, Italy; 7 Department of Clinical Chemistry and Paediatrics, Amsterdam, Netherlands; 8 Institute of Medical Genetics, School of Medicine, Cardiff University, Cardiff, UK; 9 IRRCS, Stella Maris, Pisa, Italy

**Keywords:** Barth syndrome, *TAZ* gene mutation, *In utero* cardiomyopathy, Metabolic decompensation, Lactic acidosis, 3-methylglutaconic aciduria, Gross deletions, Metabolic cardiomyopathy

## Abstract

**Background:**

Barth syndrome (BS) is an X-linked infantile-onset cardioskeletal disease characterized by cardiomyopathy, hypotonia, growth delay, neutropenia and 3-methylglutaconic aciduria. It is caused by mutations in the *TAZ* gene encoding tafazzin, a protein involved in the metabolism of cardiolipin, a mitochondrial-specific phospholipid involved in mitochondrial energy production.

**Methods:**

Clinical, biochemical and molecular characterization of a group of six male patients suspected of having BS. Three patients presented early with severe metabolic decompensation including respiratory distress, oxygen desaturation and cardiomyopathy and died within the first year of life. The remaining three patients had cardiomyopathy, hypotonia and growth delay and are still alive. Cardiomyopathy was detected during pregnancy through a routine check-up in one patient. All patients exhibited 3-methylglutaconic aciduria and neutropenia, when tested and five of them also had lactic acidosis.

**Results:**

We confirmed the diagnosis of BS with sequence analysis of the *TAZ* gene, and found five new mutations, c.641A>G p.His214Arg, c.284dupG (p.Thr96Aspfs*37), c.678_691del14 (p.Tyr227Trpfs*79), g.8009_16445del8437 and g.[9777_9814del38; 9911-?_14402del] and the known nonsense mutation c.367C>T (p.Arg123Term). The two gross rearrangements ablated *TAZ* exons 6 to 11 and probably originated by non-allelic homologous recombination and by Serial Replication Slippage (SRS), respectively. The identification of the breakpoints boundaries of the gross deletions allowed the direct detection of heterozygosity in carrier females.

**Conclusions:**

Lactic acidosis associated with 3-methylglutaconic aciduria is highly suggestive of BS, whilst the severity of the metabolic decompensation at disease onset should be considered for prognostic purposes. Mutation analysis of the *TAZ* gene is necessary for confirming the clinical and biochemical diagnosis in probands in order to identify heterozygous carriers and supporting prenatal diagnosis and genetic counseling.

## Background

The X-linked Barth syndrome (BS; OMIM #302060) is a cardioskeletal myopathy that manifests in early infancy with cardiomyopathy, hypotonia, growth delay, and neutropenia [1,2]. Barth syndrome is caused by mutations in the tafazzin (*TAZ*) gene on Xq28 and is generally associated with 3-methylglutaconic aciduria [3]. It also represents one of the neuromuscular disorders most frequently associated with left ventricular non-compaction (LVNC) [4].

Heart failure is the main cause of death in infancy followed by sepsis due to neutropenia [5], which causes some variability in the clinical course of BS [6]. Until ten years ago, mortality peaked within the first 4 years of life but over the last decade has shifted to around puberty. Improved survival is probably due to prompt diagnosis together with early and aggressive treatment of infections and cardiomyopathy [7].

BS is caused by alterations to the mitochondrial metabolism of cardiolipin (CL), a dimeric phosphoglycerolipid present predominantly in mitochondrial membranes [8,9]. CL is involved in the mitochondrial electron transport chain, playing an essential role in cellular energy metabolism, mitochondrial dynamics and the inception of apoptotic pathways [10-13]. Recently, CL has been shown to be critical for the correct organization of ATP synthase assembly [10]. CL deficiency has been detected in several tissues and cell types from BS patients [9,14,15] and it has been shown to affect both the organization and morphology of the mitochondrial inner membrane [10].

Tafazzins are phospholipid-lysophospholipid transacylases which promote structural uniformity and molecular symmetry among the cardiolipins [16,17]. Inhibition of the tafazzin pathway leads to ultrastructural and functional alteration of the mitochondria [16,18,19]. Studies carried out on a BS zebrafish model suggest that the expression of tafazzins is both tissue-specific and age-dependent, and plays an essential role in cardiac development and function [20]. A rat tafazzin knockdown model exhibits hypertrophy ventricular myocytes at birth [21]. Recently, two murine models of tafazzin deficiency were produced by inducible short hairpin shRNA-mediated *TAZ* knockdown [22,23]. Both models showed decrease of tetralineoleyl cardiolipin and accumulation of monolysocardiolipins in cardiac and skeletal muscle, associated with mitochondrial abnormalities and cardiac and skeletal muscle impairment, similar to BS [22,23].

Several *TAZ* transcript variants encoding different tafazzin isoforms have been described [16,24,25], but the function of several of them is still unknown. Four distinct tafazzin isoforms, TAZ-FL (encoding full-length tafazzin, GenBank:NP_000107.1), TAZ-Δ5 (encoding tafazzin lacking exon 5, GenBank:NP_851828.1), TAZ-Δ7 (encoding tafazzin lacking exon 7, GenBank:NP_851829.1) and TAZ-Δ5Delta;7 (encoding tafazzin lacking exons 5 and 7, GenBank:NP_851830.1) have been reported [26,27]. All four proteins are localized to the mitochondria if expressed in HeLa cells [28], but only two of them, TAZ-FL and TAZ-Δ5, have transacylase activity [16,28] with different acyl chain substrate specificity [16].

Mutations in the *TAZ* gene cause tafazzin deficiency and sequence analysis of this gene is necessary to confirm the clinical and biochemical diagnosis of BS [24,29]. The human *TAZ* gene contains 11 short exons and 10 variably long introns. At present, 105 different *TAZ* gene mutations have been reported [30,31], 94 of them associated with BS [30]. However, no correlation between the genotype and either cardiac phenotype or disease severity has been reported in BS [32]. A database of human *TAZ* gene mutations and other variants is available on-line from the Barth Syndrome Foundation [33]. Here we report five new *TAZ* gene mutations in six unrelated BS patients, including two new gross gene rearrangements.

## Methods

### Patients and controls

**Figure 1:**
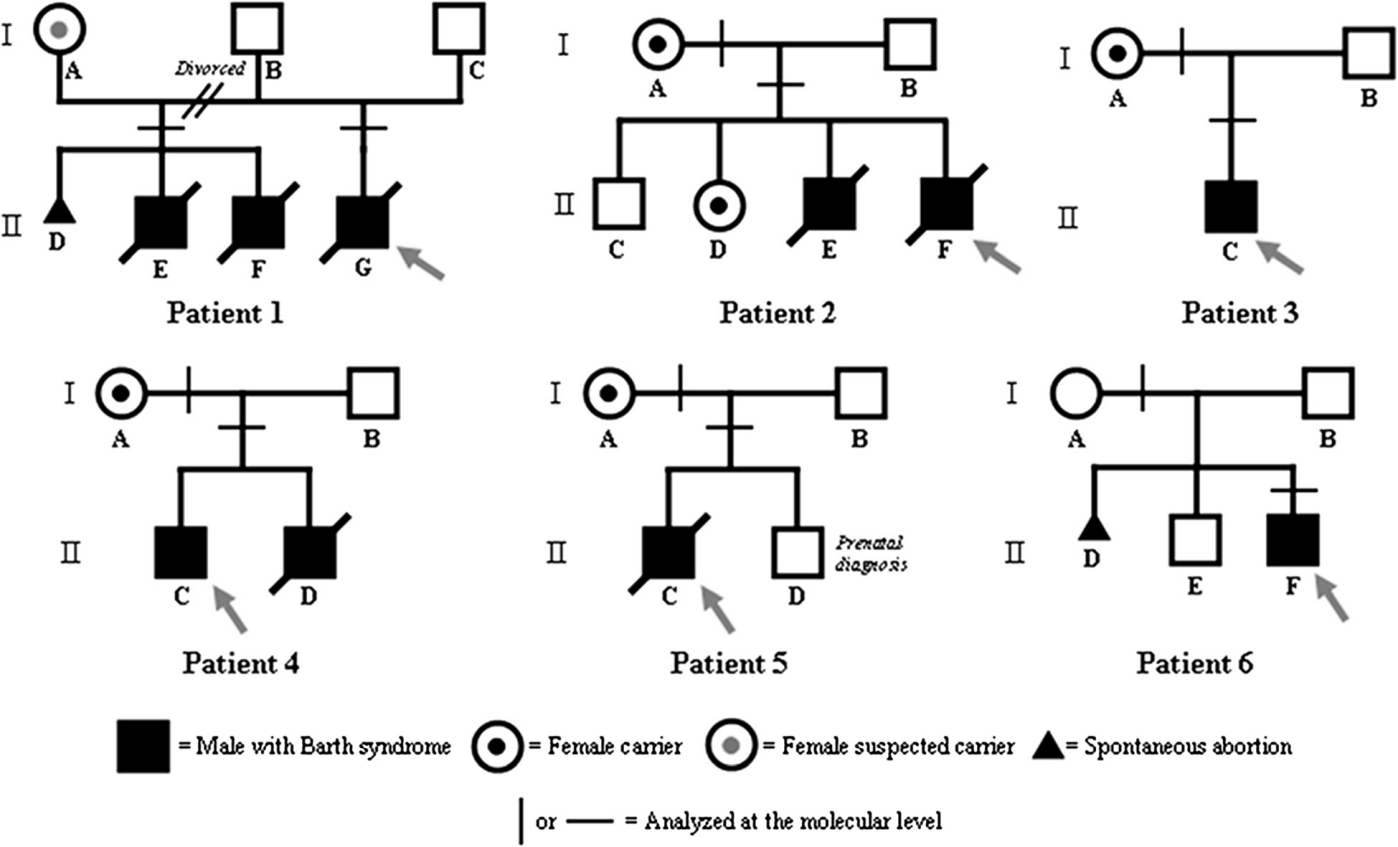
Pedigree charts of BS families.

Whole blood DNA samples from six BS patients were examined. The heterozygous carrier status of the patients’ mothers was evaluated after informed consent was obtained for all patients, in accordance with local ethical committee recommendations. The clinical histories of the patients analyzed are as follows and the pedigree charts are shown in Figure 1:

**Patient 1 (Pt1)** was the first child born to non-consanguineous parents after 36 weeks of gestation. Weight at birth was 2.25 kg with length 47 cm. On his first day of life, Pt1 manifested respiratory distress, lactic acidosis and relative neutropenia (neutrophil count, 900/mm^3^). Two days later, a severe dilated cardiomyopathy was diagnosed by heart ultrasound. Since severe hypotonia has also been presented, muscle biopsy was performed and revealed clearly reduced staining for cytochrome C oxidase, which was confirmed by a reduction in cytochome c oxidase activity measured spectrophotometrically in muscle homogenate (44% reduction in activity by reference to the lowest range). This child died 10 days after birth due to heart failure. His mother was on her second marriage. During her first marriage, she had experienced a spontaneous abortion during her first pregnancy whilst two sons had subsequently died from heart failure at the ages of 2½ months and 40 days. Suspicion of BS, and the subsequent confirmation of the diagnosis by cardiolipin/monolysocardiolipin analysis [16] (MLCL/CL ratio 253 on patient’s fibroblasts; normal 0–0.3), arose only after the birth of Pt1.

**Patient 2 (Pt2)** was born in the 39th week of gestation, with a spontaneous delivery after an uneventful pregnancy. APGAR was 1’: 8; 5’: 9; weight was 2.7 kg and length 47 cm. After 12 h of life, the newborn exhibited mild cyanosis with O_2_ Sat of 88-89%, which was moderately responsive to O_2_ administration (O_2_ Sat 92-94 %), lactic acidosis, neutropenia and dilated cardiomyopathy with left ventricular non-compaction. Biochemical analysis of urinary organic acids revealed 3-methylglutaconic aciduria. This patient died at the age of one year due to heart failure. He was the youngest of four siblings, one healthy male, one sister and a brother who had died aged 40 days due to staphylococcal pneumonia. Autopsy of this patient’s brother also revealed dilated cardiomyopathy. The clinical features of Pt2 and his family history led to suspicion of BS.

**Patient 3 (Pt3)** was the first child born with spontaneous delivery to non-consanguineous parents in the 39th week of gestation. Dilated cardiomyopathy with increased thickness of the left ventricle had been revealed during pregnancy as a result of a routine check-up. At birth, APGAR was 1’: 8; 5’: 9. Further medical investigations confirmed dilated cardiomyopathy with left ventricular non-compaction, but the patient exhibited good cardiovascular compensation. However, lactic acidosis was present and urinary organic acids analysis revealed 3-methylglutaconic aciduria, with abnormally high levels of pyruvic acid, 3-hydroxybutyric acid, methylglutaric acid, 4-hydroxyphenyl lactic acid and p-hydroxyphenyl pyruvic acid. Neutropenia was noticed, according to age appropriate ranges, on several full blood counts over a period of eight years. Diagnosis of BS was eventually confirmed by molecular and biochemical analysis (MLCL/CL ratio 70 on patient’s fibroblasts; normal 0–0.3). Pt3 exhibits intermittent neutropenia and dilated cardiomyopathy, but stable cardiovascular compensation. Occasionally, infections have occurred with fever but have been successfully treated with specific antibiotic therapies. To date, the treatment with G-CSF has not been carried out because the patient has never presented severe infections resistant to antibiotic therapy. He is currently eight years old, 20 kg (3^rd^ percentile) in weight and 115.5 cm in height (under the 2^rd^ percentile).

**Patient 4 (Pt4)** was the first child of non-consanguineous parents. Weight at birth was 2.8 kg. At the age of five months, he had a first episode of hypotonia and heart failure with gallop rhythm and his weight was under the 3^rd^ percentile. Dilated cardiomyopathy was diagnosed. Early therapeutic treatment allowed stabilization of clinical conditions. Severe neutropenia and urinary 3-methylglutaconic aciduria were also detected and confirmed during subsequent evaluations. During his life, the child has been in a good physical condition, but neurological milestones were delayed. He is currently eleven years old, 34.2 kg in weight (25^th^ percentile), 142 cm in height (25^th^ percentile) and exhibits dilated cardiomyopathy. BS was suspected and then confirmed by molecular analysis during his first physical examination, when he was 10 years old. A second son was born when Pt4 was 5 years old. The younger brother weighed 3.76 kg at birth and presented with severe sepsis at 3 days. Clinical and biochemical investigations revealed dilated cardiomyopathy, hypotonia, growth retardation, severe neutropenia and 3-methylglutaconic aciduria. At 25 months of age, he again, suffered a severe episode of sepsis due to pyelonephritis. The child died at the age of two years - ten months due to heart failure.

**Patient 5 (Pt5)** was the first child of non-consanguineous parents. He was born at 41 weeks of gestation, weighing 3.3 kg. APGAR was 1’: 9; 5’: 10 after a pregnancy complicated by gestational diabetes treated with diet therapy. Five hours after birth, he exhibited severe oxygen desaturation with cyanosis and bradycardia, requiring intubation. Hemodynamic instability was persistent with a systolic murmur of 2/6. Mild hepatomegaly was present. Echocardiography showed non-compaction of the left ventricular myocardium with reduced pump function. Blood gas analysis revealed lactic acidosis. Inotropic and diuretic therapy helped obtaining clinical and hemodynamic stabilization, followed by improvement of cardiac function. On the 8^th^ day of life, he had a first episode of cardiac arrest from which he was rescued by long resuscitation but deep coma ensued. Over the following days, clinical and hemodynamic conditions remained critical with persistence of anuria. The child died 12 days after birth. *TAZ* gene mutational analysis confirmed the diagnosis of BS in proband and the heterozygous status of the mother. A prenatal diagnosis was performed nine months later and revealed that the foetus in question did not have BS.

**Patient 6 (Pt6)** was the second child born to healthy non-consanguineous parents. Family history includes one healthy male first born and one natural abortion. APGAR was 1’: 9; 5’: 10, 3.0 kg weight, 48 cm in length. Pt6 displayed frequent regurgitation and vomiting during the first month of life with growth delay (0.1 kg per month). An episode of gastroenteritis with dehydration occurred during his seventh month of life. Further clinical investigations revealed dilated cardiomyopathy with left ventricular non-compaction, alterations in ventricular repolarization, hypokinesia, gallop rhythm, mitral and tricuspid regurgitation, and diastolic murmur 2-3/6 in the mesocardial area. Urinary 3-methylglutaconic aciduria, neutropenia and lactic acidosis were present. Acquired microcephaly with mild cerebral ventricular asymmetry and hypotonia were also evident. Cardiolipin/monolysocardiolipin analysis was performed on a dried blood spot [16] and led to the diagnosis of BS, MLCL/CL ratio was 7 (normal 0–0.3). As a consequence of a good response to therapy, at the fifteenth month of life, his physical condition was good, albeit with mild hypotrophy of the legs. He was clinically stable until two years of age at which time he presented with an episode of bronchitis and otitis with fever, which was resolved with antibiotic therapy. As in the case of Pt 3, the treatment with G-CSF has not been carried out to date, because the patient has never presented severe infections resistant to antibiotic therapy. At last follow-up he was two years old, 10.43 kg in weight (between 3^rd^-5^th^ percentile) and 86.2 cm in height (under the 3^rd^ percentile). His clinical condition is generally good with cardiovascular compensation and systolic function at the lower end of the normal range.

### Analysis of genomic DNA

Genomic DNA was isolated using the EZ1 DNA Blood 350 μl Kit (QIAGEN, Hilden, Germany). The entire coding region and intron-exon boundaries of the *TAZ* genes of the six enrolled patients were amplified and sequenced by PCR using the oligonucleotides listed in Table 1. PCR conditions are available on request. Sequencing PCR reactions were performed using the BigDye Terminator v1.1 Cycle Sequencing Kit (Applied Biosystems; Carlsbad, CA, USA) and purification was carried out using Sephadex G-50 Fine (GE Healthcare; Little Chalfont, UK). Capillary electrophoresis was performed using ABI PRISM 3130 Genetic Analyser (Applied Biosystems) as recommended by the manufacturer.

**Table 1:** **Oligonucleotides used for*TAZ*gene mutation analysis**

**Fragment name**	**Primer**	**Sequence 5’-3’**	**Included exons**	Amplified gene region*	**Length (bp)**
Taz 1-2	Taz 1-2fw	agtcaggggccagtgtctc	1 and 2	g.5224_5736	552
	Taz 1-2rv	gaaggggtttgttctgacga			
Taz 3-4	Taz 3-4fw	ctggggatatgggaagttgg	3 and 4	g.6642_7095	494
	Taz 3-4rv	ccataggtccctccaaaaca			
Taz 5	Taz 5fw	aaggtcatggggtaggaggt	5	g.7519_7714	236
	Taz 5rv	aaactcctgggcttgagtga			
Taz 6-7	Taz 6-7fw	ccccgagaatggttactgat	6 and 7	g.12983_13340	398
	Taz 6-7rv	aggcctagtctcagcacctg			
Taz 8-9	Taz 8-9fw	gggagctgaattgaactgga	8 and 9	g.13404_13753	390
	Taz 8-9rv	agacagcagacaggcagaca			
Taz 10-11	Taz 10-11fw	ggcactcctactgctcctca	10 and 11	g.14097_14513	457
	Taz 10-11rv	agcatcagtccatccctcag			

*based upon the reference nucleotide sequence NG_009634.1. Positions do not include the primer sequences.

All nucleotide positions given in this work are based upon the human *TAZ* gene nucleotide sequence [GenBank: NG_009634.1].

### Multiplex PCR analysis

Multiplex PCR analysis was performed using the primer pairs listed in Table 1. Different combinations of PCR fragments were tested in order to confirm multi-exon deletions in the *TAZ* genes of patients Pt1 and Pt2. PCR fragment combinations Taz5 + Taz8-9, Taz5 + Taz6-7 + Taz10-11, Taz5 + Taz10-11 and Taz1-2 + Taz10-11 were successfully assayed. Multiplexed PCR products were checked by agarose gel electrophoresis using 2% Agarose Low Melting (Eurobio, France) + 1% Standard Agarose (AB Analitica, Italy).

### Deletion breakpoint determination

In patients Pt1 and Pt2, who harbored large gene deletions verified by multiplex PCR, we performed long-range PCR (LR-PCR) using TaKaRa La Taq DNA polymerase (TaKaRa Bio Inc, Japan) to determine the precise locations of breakpoint boundaries. We also performed LR-PCRs using primer TAZ 5fw (Table 1) as the primer binding upstream of the deleted region, coupled with a primer binding to exon 1 of the *ATP6AP1* gene located about 7000 bp downstream of the *TAZ* gene. LR-PCR products were purified from a 0.8% agarose gel and sequenced by primer-walking [34]. The Clustal W program [35] was used to align sequences located at the breakpoints.

### Screening for the novel missense mutation p.His214Arg

The *TAZ* genes of 120 male controls were analysed by sequencing fragment Taz8-9 containing the region encoding codon 214.

### Bioinformatic analysis of interspersed repeated sequences

The *TAZ* gene region was screened for interspersed repeated sequences (SINEs and LINEs) and low complexity DNA sequences using RepeatMasker [36].

### Bioinformatic analysis of the missense mutation p.His214Arg

Tafazzin protein sequences from 13 different species (*Macaca mulatta* GenBank:NP_001028086.1, *Mus musculus* GenBank:NP_852657.1, *Rattus norvegicus* GenBank:NP_001020919.1, *Bos taurus* GenBank:DAA13200.1, *Oryctolagus cuniculus* GenBank:NP_001164847.1, *Rhinolophus ferrumequinum* GenBank:ACC68906.1, *Salmo salar* GenBank:ACN11123.1, *Danio rerio* GenBank:NP_001001814.1, *Ictalurus punctatus* GenBank:NP_001187914.1, *Aedes aegypti* GenBank:XP_001653587.1, *Drosophila melanogaster* GenBank:NP_477432.3, *Ascaris suum* GenBank:ADY45663.1, *Schistosoma japonicum* GenBank:CAX73490.1) were aligned with the human TAZ sequence (GenBank:NP_000107.1) using the Clustal W program [35]; multialignments were checked to determine the degree of evolutionary conservation of the amino acid residue (His214) involved. Further, the His214Arg amino acid substitution was also analysed by PolyPhen [37], which makes a prediction as to whether an amino acid change is likely to be deleterious to protein function [38]. Profile scores of >2.0 indicate that the polymorphism is probably damaging to protein function whereas scores of 1.5–2.0 are possibly damaging, and scores of <1.5 are indicative of a benign variant. To confirm results from PolyPhen, we used (i) the MutPred Server [39], which generates a probability for a given mutation to be deleterious to protein function (scores that range from 0 to 1), and the likelihood of it being pathogenic (scores over 0.5) [40] and (ii) SIFT [41], which yields a score of ≤0.05 if the amino acid substitution is predicted to be damaging and >0.05 if it is predicted to be tolerated.

## Results

### Identification of the TAZ mutations in six new unrelated BS patients

Molecular analysis of the *TAZ* gene in the six patients confirmed the clinical diagnosis by revealing that each one of them carried a mutated *TAZ* allele. Six different *TAZ* mutations were identified, including the previously reported c.367C>T (p.Arg123Term) nonsense mutation [42] and five new mutations: a c.641A>G (p.His214Arg) missense variant, the c.284dupG (p.Thr96Aspfs*37) microinsertion, the c.678_691del14 (p.Tyr227Trpfs*79) microdeletion and the two g.8009_16445del8437 and g.[9777_9814del38; 9911-?_14402del] large rearrangements. The *TAZ* gene variants identified in the BS patients and their related clinical features are summarized in Table 2.

**Table 2:** Genotypic and phenotypic data for the BS patients under study

**Patient**	***TAZ*gene mutation**	**Reference**	**Age of onset**	**Manifestations at onset**	**Exitus**
Pt1	g.8009_16445del8437	this work	first day of life	respiratory distress, lactic acidosis, neutropenia	ten days, heart failure
Pt2	g.[9777_9814del38; 9911-?_14402del]	this work	twelve hours of life	mild cyanosis, neutropenia, lactic acidosis, dilated cardiomyopathy	one year, heart failure
Pt3	c.641A>G (p.His214Arg)	this work	*in utero*	dilated cardiomyopathy	living, 8 years old
Pt4	c.367C>T (p.Arg123Term)	[42]	five months	hypotonia and heart failure	living, 11 years old
Pt5	c.678_691del14 (p.Tyr227Trpfs*79)	this work	five hours	oxygen desaturation with cyanosis and bradycardia, lactic acidosis	twelve days, heart failure
Pt6	c.284dupG (p.Thr96Aspfs*37)	this work	first month	frequent regurgitation and vomiting, growth delay, lactic acidosis	living, 3 years old

### Confirmation of the likely pathogenicity of p.His214Arg

The p.His214Arg amino acid substitution was not found in 120 *TAZ* alleles from healthy male controls.

**Figure 2:**
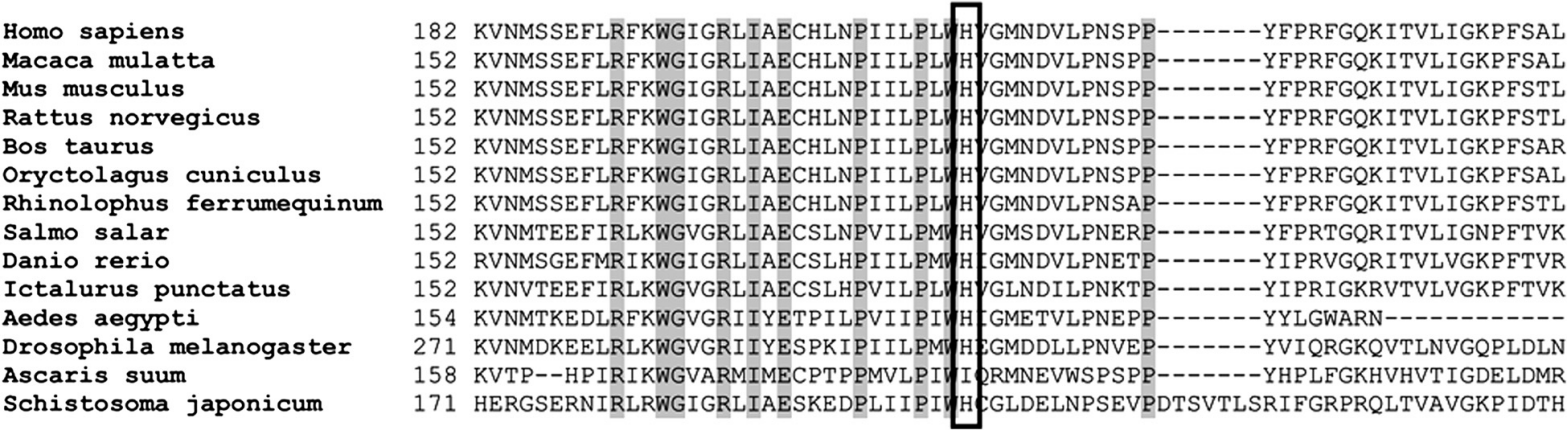
**Multialignment of the amino acid sequence of tafazzin which surrounds the new p.His214Arg substitution identified in BS patient Pt3.** Gray squares indicate amino acid residues with 100% of homology between the sequences analyzed. The His214 residue is indicated by a square.

ClustalW analysis revealed a very high degree of evolutionary conservation of histidine at position 214 (His214) in the tafazzin protein (Figure 2). Not only do all vertebrate tafazzins contain histidine at the analogous location but also do the tafazzins of two insects (*Aedes aegypti* and *Drosophila melanogaster*) and a flatworm (*Schistosoma japonicum*). This implies that this histidine residue must have been conserved for the last ~700 Ma, since prior to the divergence of deuterostomes from protostomes. Such a degree of evolutionary conservation implies a key function for this residue.

PolyPhen predicted that this mutation is probably damaging with a score of 2.862 for TAZ-FL (GenBank:NP_000107.1) and 3.123 for the TAZ-Δ5 isoform (GenBank:NP_851828.1, [25]). This prediction was confirmed by MutPred (TAZ-FL, score g = 0.732; TAZ-Δ5, score g = 0.677) and SIFT (score of 0.01 for TAZ-FL; 0.02 for TAZ-Δ5).

### Interspersed repeated sequences in the TAZ gene

Results obtained from the analysis of the human *TAZ* gene sequence (GenBank:NG_009634.1) with RepeatMasker are summarized in Table 3.

**Table 3:** **Interspersed repeat sequences within the*TAZ*gene (output data by Repeatmasker)**

	**Number of elements**	**Total length (bp)**	Percentage of sequence* (%)
Total interspersed repeats	33	7673	44.64
- SINEs:	27	6487	37.74
ALUs	25	6143	35.74
MIRs	2	344	2.00
- LINEs:	6	1197	6.96

* *TAZ* gene reference sequence NG_009634.1.

### Characterization of the large deletion breakpoints

**Figure 3:**
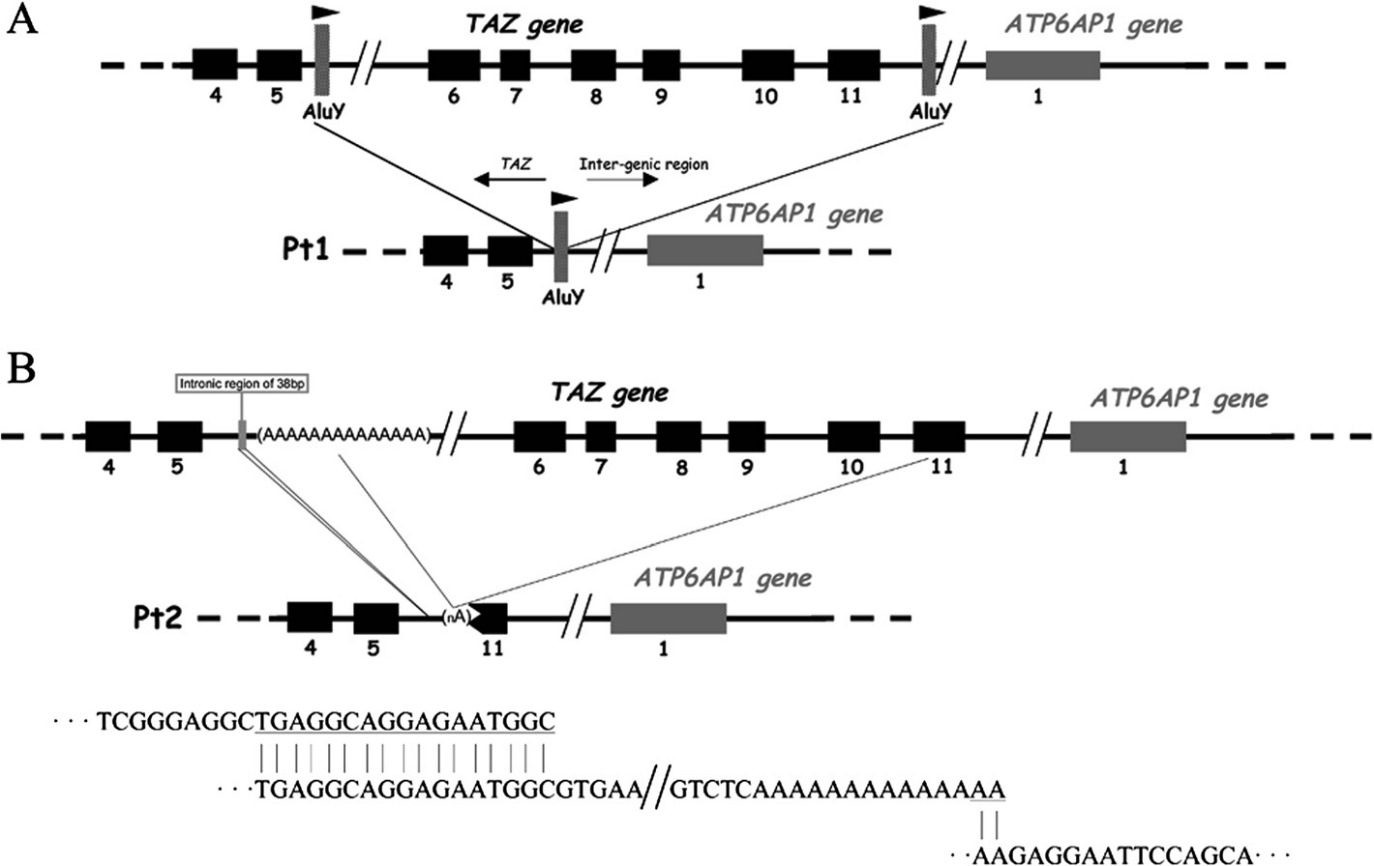
**Schematic representation of the *TAZ *deletions involving exons 6–11. A**) Relative location of *AluY* repeats downstream of the exon 5 donor splice site and downstream of the exon 11 donor splice site which are postulated to have been involved in the NAHR event causing the g.8009_16445del8437 mutation identified in Pt1. **B**) Relative locations of the breakpoint junctions of the deleted regions identified in Pt2 (one 38 bp deletion and one >4 kb deletion). Microhomologies that could have primed the sequential slippage events at the origin of g.[9777_9814del38; 9911-?_14402del] are indicated.

In patients 1 and 2, PCR analysis of the *TAZ* gene exonic sequence could not amplify exons 6 to 11. Multiplex PCR analysis was then performed, which resulted in the amplification of exons 1 to 5 but not exons 6 to 11, indicating that gross deletions spanned a large region of the *TAZ* gene in both patients. LR-PCR, performed to determine deletion breakpoints, yielded amplicons that were shorter in length than the PCR products obtained from healthy controls. Characterization of the breakpoint junctions by sequencing analysis in Pt1 revealed the deletion of an 8437 bp genomic fragment (g.8009_16445del8437) spanning exons 6 to 11 of the *TAZ* gene inclusive. The breakpoint junctions mapped to within two *AluY* repeats which were in the same orientation and located respectively 0.357 kb downstream of the exon 5 donor splice site, and 1.979 kb downstream of the exon 11 donor splice site (Figure 3A). The g.8009_16445del8437 deletion gave rise to a new hybrid *AluY* element containing the breakpoint (Figure 3A).

The analysis of the breakpoint junctions in Pt2 revealed the g.[9777_9814del38; 9911-?_14402del] complex allele. A deletion of 38 bp between nucleotides 9777 and 9814 was accompanied by a larger deletion of more than 4 kb (Figure 3B). The upstream breakpoint junction of the larger deletion is located around 2246–2260 bp downstream of the exon 5 donor splice site (nucleotides 9898_9911 in GenBank:NG_009634.1), within an A-rich sequence (14 A in the Ref Seq). One characteristic of our sequencing method was that, after a repeat structure such as a polyA stretch of >9-10 nucleotides, the sequencing signal became unreadable making it impossible to determine the number of As in the repeated sequence. Hence we could not determine precisely at which nucleotide, between 9898_9911 in NG_009634.1, the upstream breakpoint of Pt2 occurred. However, the downstream junction was located within exon 11 (nucleotide c.814).

### Determination of heterozygous carrier status

**Figure 4:**
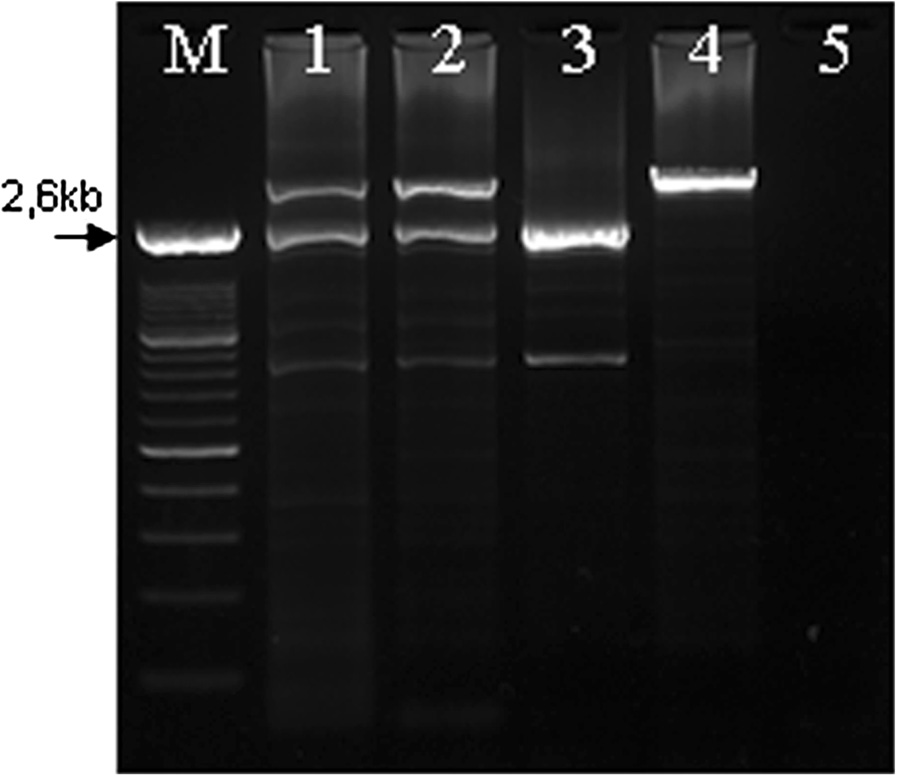
**Agarose gel analysis of the heterozygous carriers of the g.[9777_9814del38; 9911-?_14402del].** M) Marker; 1) Mother of Pt2; 2) Sister of Pt2; 3) Pt2; 4) Normal control; 5) no template DNA. Fragment of ~2.6 kb corresponds to the g.[9777_9814del38; 9911-?_14402del] allele.

Since the *TAZ* allele of Pt2 harbors a portion of exon 11, we were able to amplify across his deletion junctions with the primer pair TAZ 5fw and TAZ 10-11rv, obtaining a fragment of almost 2.6 kb. The same PCR reaction yielded a fragment of around 7 kb from the wild-type *TAZ* allele. Such LR-PCR amplification was extended to female relatives of Pt2, which allowed confirmation of the heterozygous carrier status of both his mother and sister (Figure 4).

The mother of Pt1 declined further investigation.

## Discussion

Mutations underlying human inherited disease vary greatly in size, from gross rearrangements to single base-pair substitutions. They can also originate via a number of different molecular mechanisms. Irrespective of the type of lesion, the nature, location and frequency of human gene mutations are shaped to a considerable extent, and often in remarkably predictable ways, by the local DNA sequence environment (for a review, see [43]).

Almost 45% of the genomic sequence of the human *TAZ* gene is represented by interspersed repeated sequences (SINES and LINES) and 76% of these (35% of the *TAZ* gene sequence) are *Alu* sequences. The proportion of the whole human genome that is *Alu* sequence is estimated to be around 10.6% [44]. A high proportion of *Alu* repeats within a gene sequence can promote gross gene rearrangements [45], as reported for example for the *PAFAH1B1* (LIS1) gene [46]. We report here an intragenic deletion in the *TAZ* gene, which appears to have been mediated by recombination between two *AluY* repeats. The deletion junctions in Pt1 were located within the two highly homologous and similarly oriented *AluY* repeats (Figure 3A). Non-allelic homologous recombination (NAHR) between *Alu* elements is a likely mechanism of mutation in this patient [45]. An *Alu*-mediated *TAZ* gene deletion that ablates the entire gene has also been reported [47]. These data suggest that the *TAZ* gene is located within a genomic environment that is particularly rich in repeats that can promote genetic rearrangements.

HGMD Professional v.2012.2 ( [30] update 29th June 2012) reports a total of 104 *TAZ* gene mutations of which 35.6% are missense, 12.5% are nonsense, 18.3% affect splicing, 19.2% are microdeletions, 7.7% are microinsertions and 6.7% are large gene rearrangements. Of the eight gross gene rearrangements reported, three were published [31,47,48] whereas the remaining five can be retrieved from the “Human Tafazzin Gene Mutation and Variation Database” [33] to which they were submitted directly, without supporting details. One mutation in the latter group is reported to involve the deletion of exons 6–11. In our patient sample, we identified deletions of exons 6–11 in two patients (Pt1 and Pt2) who harbored two different genetic rearrangements. Defining the exact breakpoints of such rearrangements is a prerequisite to identify heterozygous carriers of *TAZ* gene rearrangements, and for prenatal diagnosis.

Despite the high density of *Alu* repeats in the *TAZ* gene, the deletion observed in Pt2 was not mediated by NAHR between *Alu* elements. In this patient, the genetic rearrangement was found to be more complex than in Pt1 (one 38 bp deletion and one >4 kb deletion) and might have been prompted by and Serial Replication Slippage (SRS) [49]. Consistently with this assertion, the sequences flanking the breakpoints exhibit microhomologies as depicted in Figure 3B. Sequential slippage events at the 3’ end of the nascent strand during DNA replication could have been primed by these microhomologies [49]. We postulate that the SRS model, instead of the more recently proposed FoSTeS model [50], is the more likely mechanism of the rearrangement in Pt2 since the SRS model assumes that the replication slippage occurs at closely adjacent sites and gives rise to relatively small rearrangements (up to 10 kb), whereas the FoSTeS model is better suited to explain template switching that occurs over long distances, generating much larger DNA rearrangements (120 to 550 kb) [49]. Whatever the underlying mechanism, characterization of gross rearrangements in the *TAZ* gene is essential for identifying heterozygous carriers among female relatives of the probands and, as it is the case for Pt2, to confirm the carrier status of close female relatives.

In addition to these gross gene rearrangements, we identified two frameshift variants in the *TAZ* genes of our patients: a small 14 bp deletion (Pt5) and a small duplication (Pt6). Ball et al. [51] suggested that a combination of slipped mispairing mediated by direct repeats and secondary structure formation promoted by symmetric elements could account for most microdeletions and microinsertions. We analyzed the sequence environment as suggested [51], considering the 10 bp of DNA sequence flanking the c.678_691del14 deletion detected in Pt5 (ACAGTCCGCCctacttcccccgctTTGGACAGGT). This region is G/C rich and the deletion is flanked by CCGC repeats, consistently with a slipped mispairing model. At the protein level, the c.678_691del14 deletion is predicted to lead to a p.Tyr227Trpfs*79 frameshift.

The c.284dupG duplication in Pt6 (GTTGATGCGTTGgGTGAGGAGGA) might be explained by the modified slipped mispairing model [52], whereby misalignment occurs between a specific contiguous sequence on one strand and a second partially homologous, yet interrupted, sequence on the other strand. One repeat copy only becomes capable of base-pairing once an additional base has been inserted or deleted. In the case of Pt6, the first repeat is GTTGA whilst the second repeat is GTTGGA; slipped mispairing would therefore account for the duplication of a G in this first repeat. At the protein level, the c.284dupG mutation is predicted to lead to a frameshift p.Thr96Aspfs*37 with premature termination of translation.

We also detected two single nucleotide substitutions in the *TAZ* genes: c.641A>G (Pt3) and c.367C>T (Pt4). c.641A>G leads to the substitution p.His214Arg, which, although not predicted to change the polarity of the involved residue, occurs in a residue which is evolutionarily conserved, suggesting an essential role in protein function. We are unable to infer about the possible impact of the missense change directly on the three-dimensional structure of human tafazzin, as the crystal structure is not available, however molecular characterization of mutant *TAZ* alleles has been performed in a *Saccharomyces cerevisiae* model of Barth syndrome [53]. While the corresponding residue in the *S. cerevisiae* Taz1p ortholog is not conserved (it is an Alanine), the p.His214Arg mutation occurs in a putative unusual membrane anchor region in yeast Taz1p [53]. Interestingly, mutations in this membrane anchor result in two distinct biochemical fates: low fidelity sorting with increased rates of aggregation and aberrant macromolecular assembly [53].

Pt4 carried the p.Arg123Term nonsense mutation previously reported by van Werkhoven et al. [42]. According to the Human Tafazzin Gene Mutation and Variation Database [33], multiple examples of the p.Arg123Term mutation have been reported, suggesting recurrent mutation at this CpG dinucleotide mutation hotspot.

We found lactic acidosis to be present in 5/6 patients at the time they were brought to clinical attention, suggesting that, in addition to 3-methylglutaconic aciduria and neutropenia, lactic acidosis is an important biochemical marker for suspecting BS. In addition, a deep metabolic decompensation during the first hours of life, including respiratory distress and cyanosis with oxygen desaturation, was associated with the most severe outcome (Pt1, P2 and Pt5). Deep metabolic decompensation associated to these other signs may thus correlate with the severity of the disease more than it does the time of onset of cardiomyopathy that, for instance, was already detected *in utero* in Pt3 who was still alive at last follow up [54,55]. The alteration of cardiolipin metabolism in BS leads to an abnormal lipid structure of the inner mitochondrial membrane. This may result in mitochondrial leakage of metabolites, such as the lactate as we observed in our BS patients. Thus, we could hypothesize that an acute metabolic decompensation during the first hours of life, associated with respiratory distress and oxygen desaturation, or with other signs such as mild hyperammonaemia, hypoglycaemia and elevated transaminases [54] could be correlated to a deeper impairment of the mitochondrial function at the onset of BS and thus to an earlier and deeper cardioskeletal tissue damage. However, it cannot be excluded that the severity of the metabolic decompensation can be related also to other still undefined factors, as it has been reported that the pathophysiology of Barth syndrome can unlikely be explained solely on the basis of cardiolipin abnormalities and additional factors could play a role in modifying the disease expression [56]. Anyhow, these assertions remains to be proven.

Several cases of clinical manifestations of BS being detected *in utero*, as in the case of Pt3, have been reported [57,58]. Therefore, cardiolipin analysis or mutational analysis of the *TAZ* gene should be considered in cases of heart failure or sepsis during the neonatal period or of *in utero* cardiomyopathy. Such analysis should also be considered when investigating the causes of natural abortions (e.g. Pt1 and Pt6) as also previously reported [58].

Early diagnosis of BS is a prerequisite for early and effective therapy and follow-up, and for preventing crisis due to sepsis, as suggested by the clinical histories of Pt3 and Pt6. Moreover, prompt molecular characterization of BS patients is vital for prenatal diagnosis and for preventing the recurrence of BS in the same family (e.g. Pt5). Late diagnosis of BS can lead to familial recurrence of the disease as observed in the families of Pt1, Pt2 and Pt4.

## Conclusions

The human *TAZ* gene sequence is rich in interspersed repeats that may promote the occurrence of gross gene rearrangements albeit with different mutational mechanisms. Therefore, gross *TAZ* rearrangements, which may appear to be similar in different patients on the basis of ablated exons, may actually represent private mutations.

Lactic acidosis is an early biochemical marker of BS, together with 3-methylglutaconic aciduria, such as are metabolic decompensation, cardiomyopathy, heart failure and hypotonia. The severity of the metabolic decompensation at onset of BS correlates with the severity of the clinical course of the disease, and is therefore important for prognostic assessment. Cardiomyopathy may be detectable *in utero*, but its time of onset does not seem to be necessarily correlated with the severity of the prognosis.

Mutational analysis of the *TAZ* gene is necessary to confirm the clinical and biochemical diagnosis in probands, to identify heterozygous carriers and for prenatal diagnosis, but should also be considered in the presence of cardiomyopathy *in utero* and of natural abortion together with cardiolipin ratio analysis.

## Competing interests

The authors declare no competing interests.

## Authors’ contributions

LF performed the molecular genetic studies. AM coordinated the research. SM and SC contributed to the *TAZ* gene sequencing analysis of BS patients. SF performed urinary organic acid analysis of BS patients. FMV performed cardiolipin/monolysocardiolipin analysis of BS patients. LF and AM wrote the manuscript. MAD contributed to the discussion of clinical findings. DNC contributed to the discussion of genetic findings and to critical revision of the manuscript. MAD, LL, LR, EB, who are referent physicians for the patients, contributed to description of clinical cases. RG contributed to critical revision of the manuscript. All authors read and approved the final manuscript.
